# Multiplexed *tat*-Targeting CRISPR-Cas9 Protects T Cells from Acute HIV-1 Infection with Inhibition of Viral Escape

**DOI:** 10.3390/v12111223

**Published:** 2020-10-28

**Authors:** Youdiil Ophinni, Sayaka Miki, Yoshitake Hayashi, Masanori Kameoka

**Affiliations:** 1 Division of Molecular Medicine and Medical Genetics, Department of Pathology, Kobe University Graduate School of Medicine, Kobe 650-0017, Hyogo, Japan; icmrt1@gmail.com; 2 Division of Global Infectious Diseases, Department of Public Health, Kobe University Graduate School of Health Sciences, Kobe 654-0142, Hyogo, Japan; cvtokitou@gmail.com (S.M.); mkameoka@port.kobe-u.ac.jp (M.K.)

**Keywords:** HIV-1, cure, CRISPR-Cas, viral escape, *tat*

## Abstract

HIV-1 cure strategy by means of proviral knock-out using CRISPR-Cas9 has been hampered by the emergence of viral resistance against the targeting guide RNA (gRNA). Here, we proposed multiple, concentrated gRNA attacks against HIV-1 regulatory genes to block viral escape. The T cell line were transduced with single and multiple gRNAs targeting HIV-1 *tat* and *rev* using lentiviral-based CRISPR-Cas9, followed by replicative HIV-1_NL4-3_ challenge in vitro. Viral p24 rebound was observed for almost all gRNAs, but multiplexing three *tat*-targeting gRNAs maintained p24 suppression and cell viability, indicating the inhibition of viral escape. Multiplexed *tat* gRNAs inhibited acute viral replication in the 2nd round of infection, abolished cell-associated transmission to unprotected T cells, and maintained protection through 45 days, post-infection (dpi) after a higher dose of HIV-1 infection. Finally, we describe here for the first time the assembly of all-in-one lentiviral vectors containing three and six gRNAs targeting *tat* and *rev*. A single-vector *tat*-targeting construct shows non-inferiority to the *tat*-targeting multi-vector in low-dose HIV-1 infection. We conclude that Cas9-induced, DNA repair-mediated mutations in *tat* are sufficiently deleterious and deplete HIV-1 fitness, and multiplexed disruption of *tat* further limits the possibility of an escape mutant arising, thus elevating the potential of CRISPR-Cas9 to achieve a long-term HIV-1 cure.

## 1. Introduction

The presence of replication-competent integrated provirus, even under antiretroviral therapy (ART) suppression, is the main reason for recrudescent viremia in HIV-1-infected individuals. One of the most straightforward strategies to clear provirus from viral reservoirs and achieve a sterilizing cure for HIV-1 infection is to directly attack proviral DNA using gene editing toolboxes, one of which is CRISPR-Cas9. The unprecedented efficiency of CRISPR-Cas9 has elevated the platform as the most promising method to target and disrupt integrated HIV-1 DNA [1,2,3,4], including in infected primary human CD4^+^ T cells [5,6] and mouse model [7,8]. Here, Cas9 nuclease is paired with a designed guide RNA (gRNA) to recognize the complementary sequence ending with a protospacer adjacent motif (PAM)—a 20-bp spacer followed by 5′-NGG-′3 PAM in the case of *Streptococcus pyogenes* Cas9 (SpCas9) [9,10], which is widespread throughout the HIV-1 genome [2]. Among these, numerous potential target sites have been investigated. The first and most widely tested is the HIV-1 long terminal repeats (LTRs) capping both proviral ends. Cas9-induced double-strand breaks (DSBs) at both ends will theoretically excise near-whole provirus, leaving a single LTR residue, and reports initially confirmed this [5,6,11,12,13]. However, subsequent studies failed to show complete proviral excision in a consistent manner [6,14,15]. Evidently, Cas9-associated large DNA fragment excision occurred at a frequency (~27%) that was lower than expected [16], as any DSBs inside chromosomal DNA will trigger ligation of blunt ends—a DNA repair mechanism known as non-homologous end-joining (NHEJ), which occurs ~60% of the time [16,17,18]. NHEJ will nonetheless generate predictable mutations favoring single-base insertions, short deletions, or longer microhomology-mediated end-joining (MMEJ) deletions at the DSB site [18].

These findings shifted the hypothesis toward the inactivation of HIV-1 provirus by mutating functional HIV-1 coding genes via Cas9-induced DNA repair. Indeed, frameshift aberrations in essential HIV-1 genes may render viral proteins dysfunctional and inhibit productive replication. Several studies, including ours, have shown that targeted attack against HIV-1 *gag*, *pol*, *tat*, and *rev* disrupted respective protein expressions and functions, leading to failed viral replication even after latency reactivation [7,12,13,19,20]. However, prolonged infection using a replicative virion revealed a major obstacle, similar to the one faced by other anti-HIV-1 techniques including ART: The emergence of resistant viral mutants. NHEJ-induced mutations of even a single base at the proximity of the PAM site may hinder subsequent gRNA recognition and attachment to the target sequence. Thus, mutations in less vital genes may be non-deleterious to the virus, while at the same time allowing escape from repeated gRNA-Cas9 attacks [21,22]. NHEJ repair further adds to already high HIV-1 mutational escape capabilities, and indeed, this escape mechanism has been confirmed in numerous in vitro studies [14,22,23]. Strains readily develop resistance to gRNA targeting the non-coding LTR region, including the *TAR* region and primer binding site [14,15,24], highlighting the need to target genes that are essential to HIV-1 fitness. Overlapping the target of attack with RNA interference also resulted in cross-resistance [4,25]. One method to overcome NHEJ-facilitated escape is by combining two gRNAs, as tested in previous studies [26,27,28,29,30], but not all combinations have proven successful, and dual-gRNAs targeting LTR and *gag* failed to prevent HIV-1 rebound in humanized mice [31].

Here, we propose a multiplexed CRISPR-Cas9 attack of three and six gRNAs, all concentrated at HIV-1 regulatory genes as a means to inhibit acute viral replication and escape. The highly conserved *tat* and *rev* genes are indispensable as they are expressed early after HIV-1 integration and crucially dictate the success of subsequent steps in the viral life cycle [32,33]. Specifically, *tat* inhibition efficiently blocks proviral reactivation from a latent state (the ‘block-and-lock’ approach) [34], and anti-*tat* moieties have proven to strongly suppress HIV-1 replication over time in vivo [35]. We hypothesize that multiple CRISPR attacks would induce hypermutation of *tat* and *rev* genes, and escape mutations would be unlikely, as they would inflict a substantial replicative fitness cost to the virus.

## 2. Materials and Methods

### 2.1. gRNA Designs and Plasmids

Target sites inside HIV-1_NL4-3_ were identified in silico using the CRISPR web design tool (http://crispr.mit.edu) and on-target outcomes were predicted using CRISPOR (http://crispor.tefor.net). Synthetic gRNAs were cloned into pLentiCRISPRv2 (Addgene #52961, from Feng Zhang’s lab [36]) at BsmBI restriction sites, as previously described [19]. Plasmid pNL4-3, which was used to procure the HIV-1_NL4-3_ isolate, was obtained from the NIH AIDS Reagent Program. The following plasmids for all-in-one multiplexed gRNA vector development was procured from Takashi Yamamoto’s lab [37]: px330A-1x3 (Addgene #58767), px330A-1x6 (#58770), px330S-2 (#58778), px330S-3 (#58779), px330S-4 (#58780), px330S-5 (#58781), and px330S-6 (#58782).

### 2.2. Cell Culture, Transfection and Transduction

MT-4 T cells were cultured in RPMI 1640 (Nacalai Tesque) with 10% fetal bovine serum (FBS), 100 U/mL penicillin, and 100 μg/mL streptomycin, while 293T cells were cultured in Dulbecco’s modified Eagle medium (DMEM) (Nacalai Tesque) with 10% FBS. The development of stable Tat- and Rev-expressing 293T cells was described previously [19]. Vesicular stomatitis virus G (VSVG)-pesudotyped lentiviral vectors were produced by co-transfecting gRNA-Cas9-expressing pLentiCRISPRv2, psPAX2 (Addgene #12260), and pHIT/G [38] at a ratio of 3:4:1 into packaging Lenti-X 293T cells (Takara-Bio) using a FuGENE HD transfection reagent (Promega). Transduction into MT-4 cells was performed with an multiplicity of infection (MOI) of 10 (as determined previously [19]) for all single gRNA as well as the all-in-one multiplexed vectors (*tat-3*, *rev-3*, and *tatrev-6*), an MOI of 3.33 for each gRNA in *tatABC* and *revABC*, and an MOI of 1.67 for each gRNA in *tatrevABC*. To determine the lentiviral titer, a preliminary transduction assay into 293T cells was done using VSVG-pseudotyped lentivirus expressing green fluorescent protein (GFP) that was generated by co-transfecting pWPT-GFP (Addgene #12255), psPAX2, and pHIT/G, and the MOI was then determined via GFP expression (Figure S1). Cells were selected one day after transduction with 1 μg/mL of puromycin for two weeks.

### 2.3. HIV-1 Production and Infection

HIV-1_NL4-3_ was produced by transfecting 293T cells with HIV-1 DNA clone pNL4-3 with FuGENE HD. Three days after transfection, supernatants were harvested and centrifugated to clear cell debris. The HIV-1 titer was determined by measuring the virion-associated p24 protein using enzyme-linked immunosorbent assay (ELISA) according to the manufacturer’s protocol (HIV p24 ELISA kit, Rimco Corp.), whose minimal level of detection is 0.03 ng/mL. Acute HIV-1_NL4-3_ infections into MT-4 T cells were done in titers corresponding to 200 pg, 2 ng, and 20 ng of p24. Inoculated viruses were washed off with RPMI 1640 at 2 days post-infection (dpi). Once infected, the cell culture was maintained; 200 μL out of 1 ml culture medium was removed every 4 days for ELISA sampling before adding fresh medium. Cells were maintained throughout for the shorter term (16 days) assay, while cells were passaged at a 50% density every 15 days for the longer term (45 days) assay.

### 2.4. Western Blotting

Protein samples were separated by SDS-polyacrylamide gel electrophoresis (SDS-PAGE), transferred to a polyvinylidene fluoride (PVDF) membrane, blocked with 5% non-fat milk in PBS, and immunostained with the primary antibodies anti-FLAG M2 monoclonal antibody (Sigma Aldrich), CRISPR/Cas9 polyclonal antibody (Epigentek), or anti-beta-actin polyclonal antibody (BioVision). Membranes were then incubated with peroxidase-labeled secondary antibodies and visualized with Pierce western blotting substrate (Thermo Scientific) using the OptimaShot CL-420α chemiluminescence imaging system (Wako). Densitometry analysis was completed using ImageJ software.

### 2.5. On-Target Analyses

Cellular DNA was extracted from selected gRNA-Cas9-transduced cells at 16 dpi using the QIAamp DNA Blood Minikit (Qiagen). The regions stretching from the *tat* first exon and to the *rev* second exon were amplified using forward primer located in the *tat* splice acceptor (position 5777–5803 in HXB2): 5′-AAT TGG GTG TCG ACA TAG CAG AAT AGG-3′, and reverse primer in downstream of *rev* (position 8591–8606): 5′-TCC TGA CTC CAA TAC TGT AGG AGA TT-3′. The amplicons were cloned into a TA cloning vector (Mighty TA-cloning kit, Takara-Bio), Sanger sequenced (Macrogen Inc.), and aligned using GENETYX software ver. 10 (GENETYX Corp.).

### 2.6. Development of Multiplexed, All-in-One CRISPR-Cas9 Lentiviral Vector

The procedure established by Sakuma et al. [37] was adopted. Briefly, gRNAs were inserted into px330A/S vectors with *tatA* and *revA* inserted into px330A, and *tatB*, *tatC*, *revA*, *revB*, and *revC* into px330S. Step 1 of the assembly was done using the Golden Gate reaction using BpiI (Thermo Scientific), Quick ligase (NEBL), and T4 DNA ligase buffer (NEBL) before it was subjected to a thermal cycling of 3 × 37 °C for 5 min and 16 °C for 10 min and transformation. The step 2 phase of ligation was done similarly using BsaI (NEBL). The ligations of px330S-tatB and -tatC into px330A-tatA were done to create *tat-3*; px330S-revB and -revC into px330A-revA to create *rev-3*; and px330S-tatB, -tatC, -revA, -revB, and -revC into px330A-tatA to create *tatrev-6*. The clones were screened with colony PCR, and plasmids were prepared with the GenElute plasmid DNA miniprep kit (Sigma Aldrich). From here, the protocol was then modified to create lentiviral vectors. The fragment stretching from upstream of the first U6 promoter to downstream of the last gRNA (i.e., before *cas9*) was amplified from the multiplexed px330A using the following primers: 5′-TTA AGG TAC CGC TGG CCT TTT GCT C-3′ (forward) and 5′-TAG CGA ATT CTT ATG TAA CGG GTA C-3′ (reverse). The amplicon was then inserted into the lentiviral backbone LentiCRISPRv2 at Acc65I (position 1984; NEBL) and EcoRI (4202; NEBL).

## 3. Results

### 3.1. CRISPR-Cas9 Targeting tat and rev Protects T Cells from De Novo HIV-1 Infection

**Figure 1:**
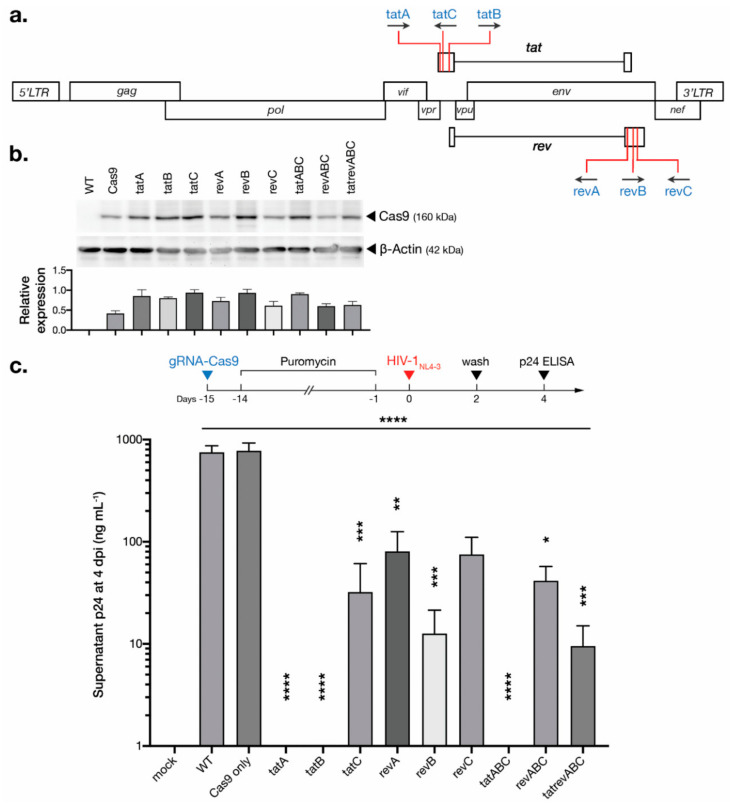
Introduction of *tat/rev*-targeting CRISPR-Cas9 protects T cells from new HIV-1 infection. (**a**) The target location of the designed gRNAs inside the HIV-1 genome. (**b**) gRNA-Cas9 constructs were transduced into T cell line MT-4 with a multiplicity of infection (MOI) of 10 and selected with puromycin for 14 days. Cells were lysed and immunoblotted with anti-Cas9 antibody and anti-β-actin as the loading control. Representative blot images and densitometry graph of Cas9 expression standardized to β-actin are shown. (**c**) As shown in the experimental timeline, gRNA-Cas9-transduced cells were challenged with HIV_NL4-3_ for 2 days before washing with fresh medium. The supernatant p24 levels were measured at four days post-infection (dpi). Assays were performed four times, and average values (±SE) are shown. One-way ANOVA applied to all groups and Dunnett’s multiple comparison test for each group compared to wild type (WT) were used to calculate significance. *, *p* < 0.05; **, *p* < 0.01; ***, *p* < 0.001; ****, and *p* < 0.0001.

We developed six SpCas9-based gRNAs targeting HIV-1 regulatory genes with three designs targeting *tat* and *rev* each, as described previously [13]. The designs were concentrated in exon 1 for *tat* and exon 2 for *rev*, where most residues integral for their respective canonical functions are located [14]. The sequence of *tatB* also coincides with *rev* exon 1, while *revA* coincides with *tat* exon 2, and all *rev* designs also target the gp41 domain of *env*. The positions of targets within the HIV-1 genome are shown (Figure 1a). Alignment against six HIV-1 subtypes (A, B, C, D, 01AE, and 02AG) revealed a high conservation of all gRNA targets as exhibited by low Shannon entropy, indicating the importance of the respective protein-coding sequences to HIV-1 genetic fitness. In silico analysis using CRISPOR (http://crispor.tefor.net) [15] predicted on-target specificity against the human genome (GRCh37), knock-out efficiency based on target nucleotide identity [16], predicted DNA repair outcomes based on flanking microhomology (which may result in larger, MMEJ-induced deletions) [17], and the probability of <3-bp indels (resulting in frameshifts) [18]. Previous CRISPR studies of the human genome have shown that the NHEJ repair of DSBs creates predictable and reproducible mutational outcomes [10,18], and thus, the selection of gRNA targets may determine the post-editing mutational profile of HIV-1 DNA and influence the risk of emerging viral escape mutants. The selected gRNA designs in this study showed relatively high specificity with reasonable probabilities of gene disruption (Table 1).

Each gRNA oligonucleotide was cloned into pLentiCRISPRv2 before transduction into T cell line MT-4 with a multiplicity of infection (MOI) of 10. Transduction of the vector containing only Cas9 (i.e., no gRNA) was also included. In this part of the study, multiplex transductions of three gRNAs (*tatABC* and *revABC*) as well as six (*tatrevABC*) were done by mixing together multiple vectors while maintaining the same overall virus titer, i.e., MOI of 3.33 and 1.67 for each vector in three and six gRNAs cocktail, respectively. The transduced cells were selected with puromycin for two weeks. The Cas9-only vector (i.e., no gRNA) was used as a control in this study, which is common in CRISPR studies [39,40], even though non-targeting gRNA might be preferable. Indeed, all transduced cell lysates expressed Cas9 properly after selection (Figure 1b), and all cell groups exhibited similar cellular growth kinetics (Figure S2). Variation in Cas9 expression was observed, however, which is most likely due to differences in transduction efficiency. Transfection, as well as downstream transduction difficulties, are common in large Cas9 vectors, and we noted similar results in our previous study [19]. Yet, several weaknesses can be identified in our methodology, such as the absence of ultracentrifugation or spin-column concentration of pseudovirus, or the relatively large amount of lentiviral volume (~100 μL) needed to achieve an MOI of 10, which might have caused inadequate virus-cell interaction [41].

We have previously shown that CRISPR-Cas9 abolished viral replication in chronically infected T cells, in both persistent and latent infection models [19]. In the current study, we tested whether gRNA-Cas9 introduction can protect MT-4 T cells—one of the most susceptible cell lines to HIV-1 infection [42,43]—from acute de novo viral challenge (i.e., an inhibition strategy as opposed to inactivation, as reviewed by [3]). Wild type (WT), Cas9 only (Cas9), and gRNA-Cas9-transduced MT-4 T cells—either in single, or in a cocktail of three or six gRNAs—were challenged with replicative HIV-1_NL4-3_ in titer corresponding to 200 pg of p24 protein. A low infecting titer was selected to observe potential viral escape from infected yet surviving cells from the highly cytopathic HIV_NL4-3_ isolate. The target sequences within the *tat* and *rev* genes of HIV-1_NL4-3_ harbor zero mismatches against all gRNA designs. The cells were washed and transferred to a fresh medium at 2 dpi, and the supernatants were collected from all cell groups at 4 dpi. An ELISA measurement at 4 dpi showed a significant decrease in HIV-1 p24 production in all T cells equipped with gRNA-Cas9 (Figure 1c). Cells transduced with *tat*-targeting gRNAs exhibited the highest reduction of p24 compared to the unprotected WT T cells, with *tatA-*, *tatB-*, and *tatABC*-transduced cells producing near undetectable levels of p24. Both *revABC-* and *tatrevABC*-transduced cells, however, showed a less HIV-1 inhibitory capacity than even single constructs, which might be due to the lower MOI for each gRNA—particularly potential ones, e.g., *tatA* and *tatB*—in cocktail transduction, where there were only one-third of the original MOI for each gRNA for *revABC* and one-sixth for *tatrevABC*. It is important to note that we have previously shown these *tat*- and *rev*-targeting gRNA-Cas9 constructs to abolish the expression and function of respective Tat and Rev proteins, supporting the notion that HIV-1 suppression was achieved because of *tat/rev* gene disruption [19].

### 3.2. CRISPR-Cas9 Targeting tat Inhibited HIV-1 Escape Observed for All Other gRNAs

**Figure 2:**
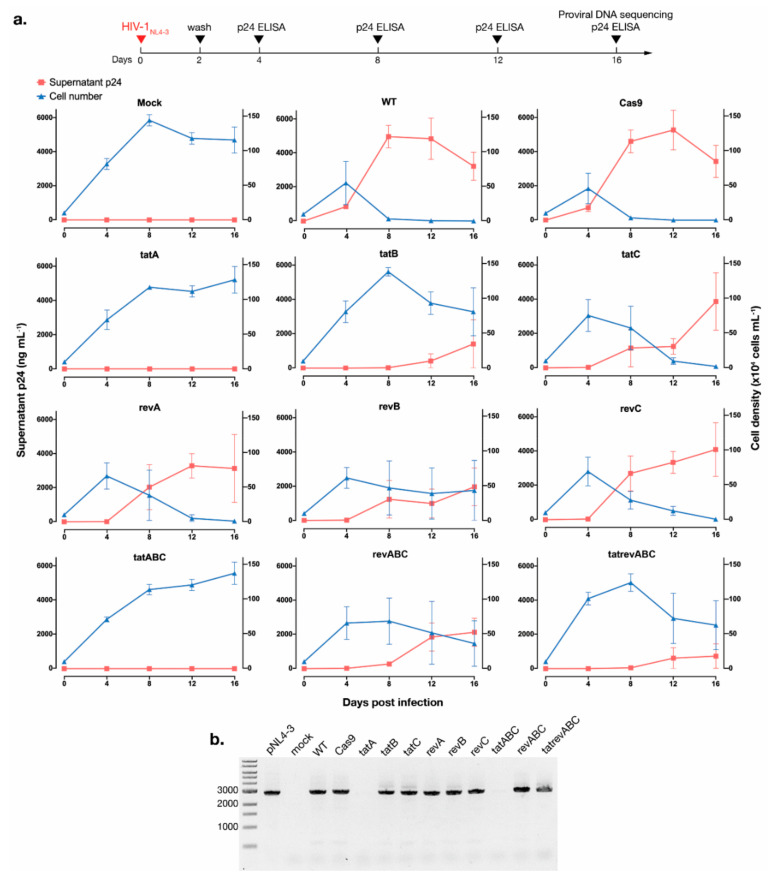
CRISPR-Cas9 targeting *tat* inhibited acute HIV-1 escape. (**a**) As shown by the timeline at the top, T cells were infected with HIV-1_NL4-3_ equivalent to 200 pg of p24 and cultured for 16 days, and supernatants were collected every four days for p24 measurement. To measure HIV-1-induced cytopathic effects, viable cells were counted with trypan blue exclusion every four days. The kinetics of supernatant p24 are shown by the red line (left y-axis) and cell density by the blue line (right y-axis). Assays were performed three times with average values (±SE) shown here. (**b**) Cellular DNA was extracted at 16 dpi and ~3 kb amplicons between *tat* exon 1 and *rev* exon 2 were amplified for each cell group. Amplicons were analyzed using gel electrophoresis, and a representative gel image is shown.

Cultures of CRISPR-transduced T cells once exposed to infectious HIV-1_NL4-3_ were incubated for 16 days. From each culture, 200 μL of supernatants was collected every four days for p24 measurement, while all cells were maintained in the culture throughout. Viral escape—as indicated by increased p24 in the supernatant—was apparent for almost all gRNA-Cas9 samples as early as 8 dpi, including that of multiplexed *revABC*-transduced cells, although p24 productions in all samples were visibly delayed compared to that of the WT and Cas9-transduced cells (Figure 2a, red line, left y-axis). However, T cells transduced with *tatA*, *tatB*, *tatABC*, and *tatrevABC* managed to maintain suppression of viral p24 up to 16 dpi. Remarkably, the *tatA* and *tatABC* transduction resulted in close to zero p24 production for the entire duration—a finding similar to a previous report using multiplexed *gag*- and *tat/rev*-targeting gRNAs done by the Berkhout group, albeit in a much shorter time span in this study [26]. Multiplexed *tatrevABC* transduction, however, failed to protect T cells from viral breakthrough, presumably due to a lower MOI for each gRNA in the lentiviral cocktail. It is worth noting that while p24 kinetics is used in other studies with similar objectives [14,23,26], our assay did not quantify normalized HIV-1 RNA or RT activity, which would be a more precise surrogate of replication breakthrough.

As a highly cytopathic infectious clone [44], HIV_NL4-3_ rapidly caused cell deaths in almost all cell groups after 8 dpi. Exceptions were observed for *tatA* and *tatABC*-transduced cells, both of which exhibited growth kinetics comparable to that of mock-infected cells (Figure 2a, blue line, right y-axis). Cellular DNA at 16 dpi was extracted from all cell groups, and a ~3-kb region between an upstream of *tat* exon 1 and downstream of *rev* exon 2 was amplified (Figure 2b). While no bands were visualized for *tatA* and *tatABC* samples, 3-kb amplicons were detected for almost all samples, indicating the presence of provirus whose region from *tat* exon 1 to *rev* exon 2 was not excised. Particularly, no smaller bands were apparent in *tatrevABC*, indicating a negligible excision of the region between DSBs in *tat* and *rev*, even though short excised products are preferred in conventional PCR amplification. Thus, Cas9 cleavage at two sites would not necessarily lead to a whole excision of the region in between, but mutations should be expected at DSB sites if editing is successful—further establishing as a proof-of-concept that instead of cleaving LTRs at both ends to excise provirus, a disruption of essential coding HIV-1 genes should give a more consistent outcome.

### 3.3. Cas9-Induced Mutational Pattern of HIV-1 Provirus after Prolonged Acute Infection

**Figure 3:**
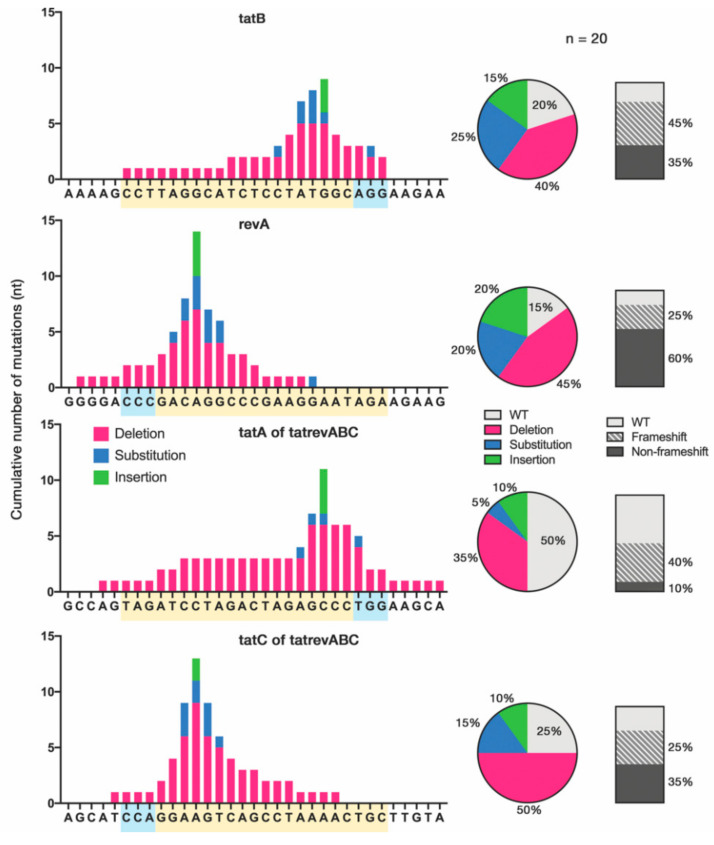
Mutational pattern in gRNA-Cas9 target sites. On-target sites were amplified from cellular DNA of *tatB*, *revA*, and *tatrevABC*-transduced cell groups at 16 dpi. The *tat* and *rev* genes were amplified and TA-cloned to produce 20 samples for each target site, followed by Sanger sequencing. The cumulative number of mutations for each nucleotide is shown in the bar graph (left). Mixed substitutions with deletion or insertion were grouped as deletion or insertion, respectively. Yellow boxes indicate the gRNA sequence, and blue boxes indicate the PAM site. The proportion of mutation types and frameshift mutations for each gRNA are shown by the pie charts and vertical slices, respectively.

The varying extent of HIV-1 inhibition may be explained by the heterogeneity of NHEJ-induced mutations caused by each gRNA-Cas9 construct. To compare the mutational pattern accumulated after prolonged acute infection between cell groups, cellular DNA was extracted from infected cells at 16 dpi, and the *tat* and *rev* genes were sequenced. On-target sites were amplified from the cellular DNA of *tatB*- and *revA*-transduced cells, while the *tatA* and *tatC* target sites were amplified from the multiplexed *tatrevABC*-transduced cells. Here, multiple indels as well as substitutions concentrated at the Cas9 cleavage site—3-nt upstream of the 3′-NGG-5′ PAM—can be observed (Figure 3, see Figure S3 for details). Several groups, including ours, have found that Cas9-induced mutations in HIV-1 DNA manifest mostly as deletions [14,19,26], and a recent study reported single insertions and short deletions as the overwhelming majority of reproducible NHEJ-mediated mutations [18]. Yet, we observed more base substitutions than was expected (20–25% in the single gRNA *tatB* and *revA*), which implies that escape patterns manifesting as substitutions may theoretically inflict less damage on HIV-1 replication fitness. It is worth noting that most *revA*-induced mutations did not lead to frameshifts, and HIV-1 readily escaped from *revA* transduction (Figure 2a), which again indicated how non-deleterious mutations facilitate viral evasion from CRISPR-Cas9 as confirmed in previous studies [14,21]. Nevertheless, this result was obtained by the Sanger sequencing of a limited number of TA clones, which poorly elucidates the link between genotypic pattern and viral phenotypes; analysis using on-target next-generation sequencing (NGS) (e.g., [14]) is warranted. Half of *tatA* sequences also showed a high number of WT sequences, and this may be due to the lower MOI of *tatA* transduction (1/6 of *tatA* single gRNA) in the cocktailed multiplex method in this part of the study.

### 3.4. Cas9-tat gRNAs Maintained HIV-1 Suppression after 2nd Round Infection and Co-Culture with Unprotected T Cells

**Figure 4:**
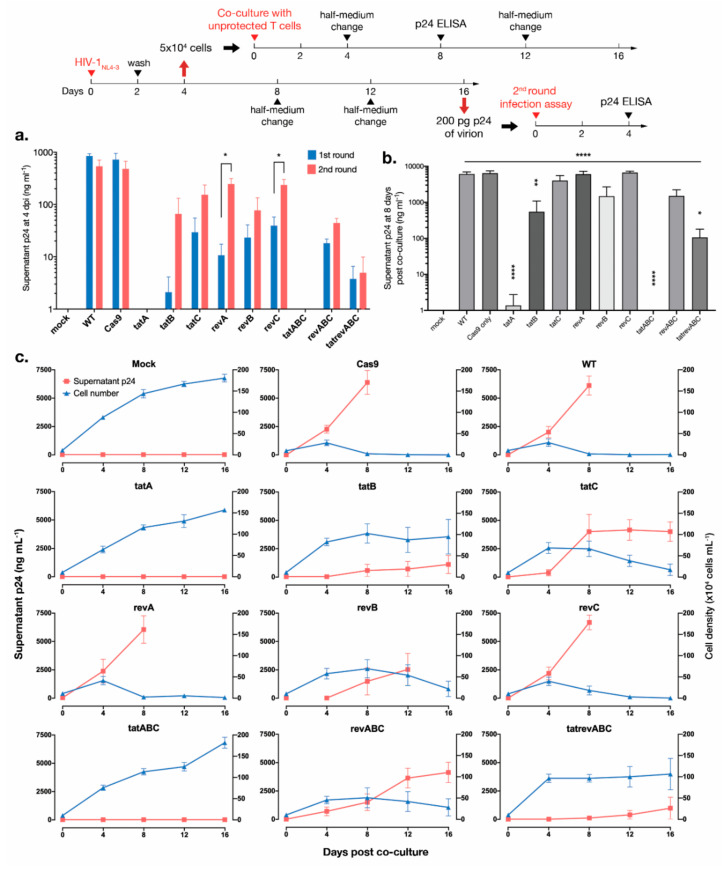
HIV-1 inhibition by *tat*­-targeting CRISPR-Cas9 was maintained after a second round of infection and co-culture of infected cells with unprotected T cells. (**a**) As shown by the timeline, T cells were first infected with HIV-1_NL4-3_ equivalent to 200 pg of p24 and cultured for eight days. The supernatants were collected and virions equivalent to 200 pg of p24 were used on an infected to an uninfected batch of the same group of cells to represent the second round of infection. The supernatant p24 levels at 4 dpi were measured and compared to samples from the first round of infection at the same time point. The assays were performed three times with average values (±SE,) as shown. An unpaired t-test was done to identify significant differences. *, *p* < 0.05. (**b**) At 4 dpi of the first round of infection, infected cells (5x10^4^) were collected, washed two times with fresh medium, and co-cultured with unprotected, wild-type MT-4 T cells (5x10^4^). The supernatant p24 levels were measured at eight days post-co-culture. The average values (±SE) of three assays are shown. One-way ANOVA applied to all groups and Dunnett’s multiple comparison test for each group compared to WT were used to calculate significance. *, *p* < 0.05; **, *p* < 0.01, and ****, *p* < 0.0001. (**c**) Co-cultures were maintained until 16 dpi. The kinetics of supernatant p24 levels are shown by the red line (left y-axis), and viable cells as counted with trypan blue exclusion are shown by the blue line (right y-axis). The average values (±SE) of three assays are shown.

To confirm whether Cas9-induced HIV-1 mutation causes any phenotypic change in virion infectivity and cytopathic effects, we conducted two follow-up assays. First, to investigate the released virions, a 2nd round infection assay was performed whereby the supernatants of infected cells at 16 dpi were collected, p24 levels were determined, and a virion equivalent to 200 pg of p24 was used to infect a fresh, uninfected batch of cells from the same groups. Mutated virions with sufficient replication fitness were expected to promptly escape from gRNA-Cas9 disruption, and thus delays in viral replication, as seen during previous analysis (Figure 2a), would be diminished. The levels of p24 at 4 dpi were measured and compared between the 1st round of infection with HIV_NL4-3_ produced from pNL4-3 transfection and the 2nd round of infection with viruses harboring accumulated Cas9-induced mutations. Increased p24 levels in the 2nd round were observed for *revA*- and *revC*-transduced cells, indicating that HIV-1 mutants readily escaped from subsequent gRNA-Cas9 targeting (Figure 4a). This effect was not apparent for other sample groups and both *tatA* and *tatABC* essentially block viral infection completely. The 2nd round of infection, however, was not done in parallel to the 1st round using WT HIV_NL4-3_,­ and thus may not have elucidated the actual replication fitness of these mutant viruses. Nevertheless, a comparison between the 1st and 2nd round of replication for each gRNA suggests that escape mutants produced after prolonged gRNA-Cas9 pressure may evade subsequent targeting with the same gRNA or, in this case, acquire an enhanced replicative capacity in a fresh round of infection to the same gRNA-Cas9-transduced cells.

Secondly, to examine any phenotypic change of cell-associated HIV-1, 1st round infected cells at 4 dpi were collected, washed, and co-cultured together with the same amount of unprotected WT T cells (Figure 4b). As shown in Figure 2a, HIV-1_NL4-3_ showed high cytopathic activity towards WT cells after 8 dpi, and thus a short length of incubation time of four days was chosen. Eight days after starting co-culture, supernatants were collected, and p24 levels were measured. Remarkably, *tatA* and *tatABC*-transduced cells once again showed undetectable residual viral replication, indicating that protection against HIV-1 was maintained sufficiently so that no productive cell-associated transmission could occur from these once-exposed cells. Several other gRNAs also inhibited viral transmission to a varying degree, while *tatC*, *revA*, and *revC* gRNAs failed to protect unprotected T cells and p24 levels surged to those comparable to that of the WT. Co-cultures were maintained until 16 dpi, from which contrasting supernatant p24 and T cell growth kinetics can be observed in *tatA* and *tatABC* against all other gRNAs (Figure 4c).

### 3.5. Development of Multiplexed tat/rev-Targeting CRISPR-Cas9 in an All-in-One Vector

**Figure 5:**
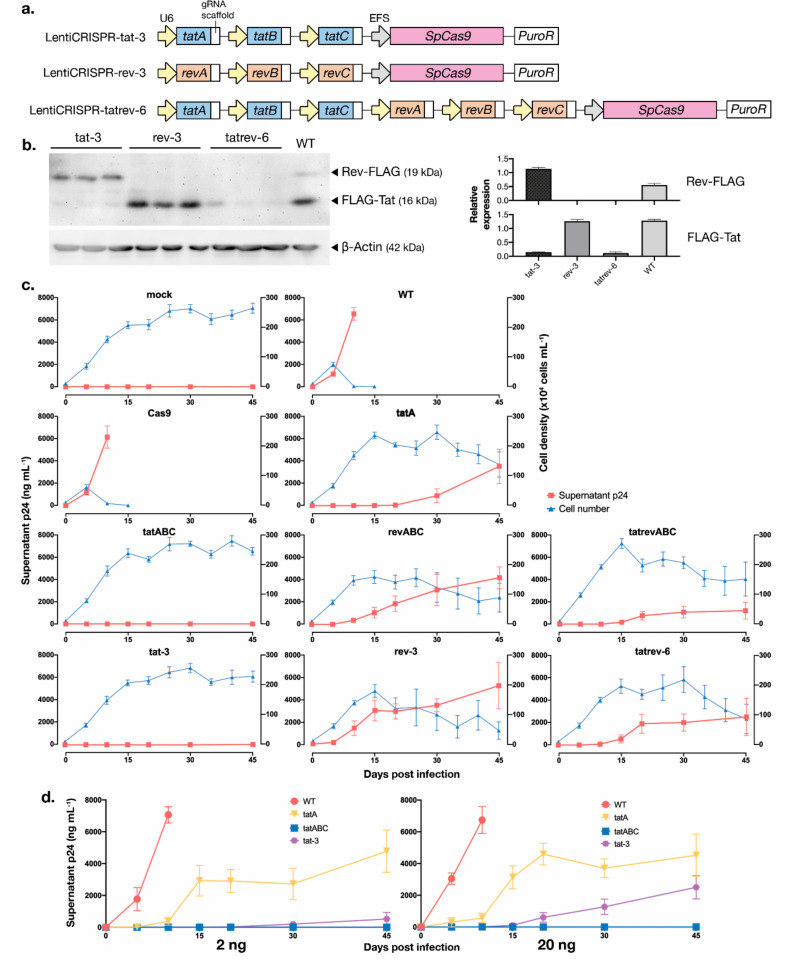
Multiplexed *tat/rev*-targeting all-in-one vectors and their HIV-1 inhibition properties. (**a**) Schematics of vectors harboring three *tat*-targeting (lentiCRISPR-tat-3), three *rev*-targeting (lentiCRISPR-rev-3), and six *tat*- and *rev*-targeting gRNAs (lentiCRISPR-tatrev-6). Multiplexed gRNAs from px330A were cloned into plentiCRISPRv2. (**b**) Functional assay of the developed multiplexed vectors; three plasmid clones for each *tat-3*, *rev-3*, and *tatrev-6* were tested. The transfections were done for three days into transformant 293T cells, which stably express Tat and Rev proteins. The cells were lysed and immunoblotted with anti-FLAG and anti-β-actin as a loading control. The representative blot images and densitometry graph of FLAG-Tat and Rev-FLAG expression standardized to control are shown. (**c**) gRNA-Cas9-transduced T cells were infected with HIV-1_NL4-3_ equivalent to 200 pg of p24 and cultured for 45 days. The assay was not performed in WT or Cas9-only cells from 15 dpi onward, as extensive cell deaths were apparent. The supernatants were collected at selected time points (5, 10, 15, 20, 30, and 45 dpi) for p24 measurement, and the cell density was determined with trypan blue exclusion every five days. The assays were performed three times with the average values (±SE) shown here. (**d**) Similar assays were done with an increased titer of HIV_NL4-3_: equivalent to 2 ng and 20 ng of p24, with selected gRNA groups. Average values (±SE) of three replicates are shown.

While multiple analyses so far have shown the strong HIV-1 inhibitory properties of *tatA* and *tatABC*-Cas9, it is of concern that the six-gRNA multiplexed lentiCRISPR *tatrevABC* failed to exhibit any satisfactory results. Multiplexing up to this point has been done by mixing multiple gRNA-Cas9 vectors in a lentiviral cocktail, and the expressions of all vectors were selected with the same antibiotic. We hypothesize that this method may produce inconsistent and variegated expression of gRNAs in the target cells. By fitting all gRNAs into one vector, a uniform integration process into cells would ensure the expression of all gRNA-Cas9s in a more consistent and homogeneous manner after selection. To achieve this, we designed a lentivirus vector containing three or six gRNA expression cassettes in an all-in-one vector, using a modification of a method established by Sakuma et al. [37]. Here, the U6-driven px330A vector was modified using the Golden Gate assembly method, which allows for the incorporation of two to seven gRNAs [37,45,46]. We developed three constructs to target either *tatA*-to-*C* (*tat-3*), *revA*-to-*C* (*rev-3*), and all six targets inside *tat* and *rev* (*tatrev-6*) (Figure 5a). The constructs were screened using colony PCR (Figure S4). We then amplified regions stretching from the first U6 promoter to the last gRNA scaffold and cloned them into lentiCRISPRv2 before transfection into packaging cells to create one lentiviral vector harboring multiple gRNAs. A functional screen was conducted by transfecting three replicates for each plasmid into 293T cells stably expressing Tat and Rev proteins, as explained previously [19]. The knockdown of Tat, Rev, or both Tat-and-Rev expression by *tat-3*, *rev-3*, and *tatrev-6*, respectively, were confirmed by immunoblotting (Figure 5b).

CRISPR-transduced T cells were then infected with HIV-1_NL4-3_ equivalent to 200 pg of p24 in a way similar to the analysis used to produce Figure 2, but the cell cultures were continued for up to 45 dpi (Figure 5c). Here, the all-in-one *tat-3* construct managed to maintain suppression until 45 dpi, which is not inferior to the results using the multiplexed multi-vector *tatABC*. An increase of p24 was noted for the single gRNA *tatA* starting from 30 dpi. Escape was also observed for the multiplexed multi-vector *revABC* and *tatrevABC*, as well as the all-in-one vectors *rev-3* and *tatrev-6*. While *rev* targeting mostly failed to protect T cells from prolonged infection, as shown in the previous analyses, *tatrev-6* was expected to exhibit strong inhibition properties. Nevertheless, HIV-1 escape readily manifested in the *tatrev-6* cell group, and the result was even relatively inferior to that using *tatrevABC*. The insertion of a much larger vector of six gRNAs (overall plasmid size of ~15.5-kb, as compared to ~14-kb for 3 gRNAs and ~13-kb for single gRNA) may disrupt lentiviral construction and transduction efficiency, and the titer of produced lentivirus was notably lower in *tatrev-6* (Figure S5). This problem may be circumvented by using episomal vectors, e.g., adeno-associated virus-9 (AAV_9_) [47]. Moreover, multiple identical U6 promoters for each gRNA may lead to self-recombination, as shown in shRNA [48] and CRISPR-Cas9 constructs [49], which might have further limited the efficiency of *tatrev-6*. However, preceding assays have shown that *rev*-targeting constructs were ineffectual nonetheless, so we saw no benefit of pursuing optimization of *tatrev-6* further and instead focused on a *tat*-targeting construct.

Finally, a similar assay was conducted with higher titers of HIV_NL4-3_ in the initial infection: Equivalent to 2 ng and 20 ng of p24. Most remarkably, *tatABC* managed to maintain viral suppression after 20 ng of HIV_NL4-3_ infection, i.e., 100 times the initial assay, for up to 45 dpi (Figure 5d, kinetics of cell density are shown in Figure S6). By contrast, an increase in p24 was noted for *tat-3*, confirming that the escape inhibition may be HIV-1 infection dose-dependent.

In summary, multiplexed *tatABC* managed to protect T cells from acute infection of replicative HIV_NL4-3_ for up to 45 dpi, from viruses in a 2nd-round infection assay, in co-culture with unprotected T cells, and from an increased HIV_NL4-3_ infection-dose to 20 ng of p24. This effect is superior to all single gRNAs, multiplexed *revABC,* and *tatrevABC*. The newly developed all-in-one vector *tat-3* exhibited non-inferior viral inhibition properties for up to 45 dpi but failed to do so at an increased infection-dose.

## 4. Discussion

Analogous to its capability to escape from ART and other gene editors [50,51], HIV-1 is capable of generating escape mutants to evade direct HIV-1 provirus disruption by CRISPR-Cas9. The combination of up to two gRNAs to simultaneously attack targets inside HIV-1 DNA has been tested previously [5,26,27,28,29,30]. However, potent inhibition of viral escape is elusive, and not all combinations of gRNAs can successfully achieve this feat. A substantial number of HIV-1 proviral-targeting CRISPR studies have been done by the Berkhout group [22,25,26,30,52], who identified gRNA pairs targeting *gag* and *tat/rev* first exon as a strong inhibitor of HIV-1 escape for up to 128 days, with the latter gRNA partially overlaping with the *tatB* sequence in this study. It is certainly of interest to identify predictors of potent gRNAs to suppress viral escape, but it remains unclear which factor is the most contingent: specificity of gRNAs, on-target disruption and MMEJ-mediated collateral damage, accessibility of DNA cleavage sites, or sequence conservation within HIV-1 [15,29,53]. In silico on-target analysis (Table 1) did not seem to predict the high efficacy of *tatA* in this study. Viral escape was reported to correlate with the distance between gRNA sites in multiplexed targeting [26], but we noted viral escape for *tatrevABC* (~3-kb distance in between). Nevertheless, from this study result, we propose that inactivating HIV-1 coding genes with inherently vital functions is key to suppressing viral escape, as it inflicts a substantial fitness cost on the virus. The early-expressed HIV-1 Tat is an essential regulator of transcription and is the sole viral factor involved in the ­“block-and-lock” cure strategy, as an absence of Tat drives HIV-1 latency [34].

Introducing more gRNAs will naturally increase the potency of HIV-1 provirus disruption, e.g., quadruplex gRNAs confer stronger HIV-1 suppression than duplex gRNAs [8]. Dual gRNA targeting also produced fragment inversion, albeit less frequently than target site mutation [52] and this further promotes HIV-1 DNA hypermutation. However, multiplexing gRNA also translates to a multiplied risk of off-target damage to the host DNA. Previous studies reported the absence of Cas9-induced off-target damage, including in endogenous LTRs [14,19,37,54], but most studies used a single gRNA, and this risk is much less known in vivo. Paired nickases have been proposed to increase gRNA specificity, and the multiplexing system used in this study may also incorporate Cas9 nickases [37,55]. On-target risk is also concerning since an unexpectedly large on-target deletion of up to 2.3 kb and chromosomal rearrangements have been reported in Cas9-mediated knockdown in mitotically active cells [56,57]. This may hamper the application of CRISPR-Cas9 to target human genes, e.g., *CCR5* [58], but may further provide a rationale for targeting foreign viral genes—preferably in regions distal to HIV-1 integration junctions—analogous to the original functions of CRISPR-Cas in bacteria loci.

A one-base mismatch in a target sequence may negatively affect CRISPR editing outcomes, which is problematic in the case of HIV-1 due to its highly rapid mutational trait. While this study only tested antiviral activity against HIV-1_NL4-3_, the Berkhout group has analyzed combinatorial gRNA attacks against several HIV-1 isolates, which demonstrated a robust viral inhibition over time if provided with stringent base-pairing. Moreover, NGS studies from peripheral blood mononuclear cells (PBMCs) of HIV-1-infected individuals revealed the possibility of a one-gRNA design to target all quasispecies, which is further limited by prolonged ART pressure [29,59]. These studies targeted LTR, *pol*, and *gag*, while *tat* sequences—including the target sites in this study—also display high conservation across diverse HIV-1 subtypes [19]. Nevertheless, the sheer breadth of HIV-1 genetic diversity makes it unlikely for viral target sequences to be covered by one or several predetermined gRNA designs. A better approach is to characterize the HIV-1 population in an individual patient before designing the gRNA, i.e., the personalized gRNA approach [60], and we propose multiplexed Cas9 targeting of the *tat* variant from the identified quasispecies sequence [61] to achieve long-term viral suppression. Experiments with multiple HIV-1 clades and primary strains are warranted.

Several methodological shortcomings from this study should be acknowledged. Ultracentrifugation or viral precipitation could be done to increase pseudovirus concentration. Although frequently overlooked in studies, the expression of gRNAs and target mRNAs should be confirmed in the transduced cells (e.g., via Northern blotting), particularly in the case of multiplexed editing. HIV-1 RNA quantification and on-target NGS could more accurately represent the extent of viral replication inhibition and proviral mutations. The usage of non-targeting gRNA and specific titration using GFP-bearing lentiCRISPRv2 [62] could give a more controlled outcome and we implore CRISPR-Cas9 studies to include these to better clarify editing results.

Furthermore, other technological upgrades may give a superior outcome and safety profile to approach the clinical application of anti-HIV CRISPR-Cas9. High-fidelity SpCas9 variants (e.g., evoCas9) may exhibit a better on-target specificity [63]. Different Cas9 orthologs provide different PAMs, which broadens target possibilities—*Staphylococcus aureus* (SaCas9), in particular, has been widely tested and gives better flexibility in vector loading due to its smaller size [7,8,31,64]. To overcome the self-recombination of U6 promoters, several alternatives in multiplexing gRNAs are available [65,66,67,68], even up to 25 gRNAs with CRISPR-Cas12a [69]. Cas12a also cleaves DNA at sites distal to the PAM recognition site, which allows for retargeting and reduces the possibility of NHEJ-mediated HIV-1 escape [70], and a recent study reported full infectious HIV-1 inactivation with just one crRNA design [71]. A Tat-mediated switch is another ingenious method to achieve selective Cas9 expression in HIV-1 infected cells [72,73]. Moreover, transgene delivery remains a major barrier of in vivo gene editing. Lentiviruses, as used in this study, may heighten risks of aberrant DNA integration and carcinogenesis, while several HIV-1-related studies have tested safer techniques, e.g., AAV_9_ [7,31], piggyBac transposon [5], nanoparticles, and microvesicles [74,75], to introduce gRNA-Cas9. Cell-based delivery using stem cells and CAR-T cells [76] is merited for investigation. Finally, future studies should test HIV-1-targeting multiplexed gRNA-Cas9 in ex or in vivo model by utilizing primary CD4+ T cells or humanized mice [77].

In conclusion, this study reaffirmed previous study results where multiplexed CRISPR-Cas9 proviral disruption successfully inhibited acute HIV-1 rebound in vitro. We further proposed a concentrated attack on the *tat* gene as a promising means to block viral escape and tested novel all-in-one vectors harboring multiple gRNAs against replicative HIV-1. The combination of ART transformed HIV/AIDS from a deadly disease to a chronic disease, and the next step is to render it curable. The combination of gRNAs targeting HIV-1, concerted with ART and other anti-HIV modalities, may elevate CRISPR-Cas9 as an encouraging treatment approach to achieve long-term HIV-1 cure.

## Figures and Tables

**Table 1:** gRNAs used in this study and their predicted outcomes.

gRNA	Target Sequence + PAM ^a^	Position in HXB2	Shannon Entropy ^b^	Specificity ^c^	Knock-out Efficiency ^c^	MMEJ deletion ^c^	Frameshift^c^
tatA	TAGATCCTAGACTAGAGCCCTGG	5840–5862	0.14	76	39	61	77
tatB	CCTTAGGCATCTCCTATGGCAGG	5954–5976	0.06	91	53	89	74
tatC	CCAGGAAGTCAGCCTAAAACTGC	5891–5869	0.22	81	35	58	79
revA	CCCGACAGGCCCGAAGGAATAGA	8415–8393	0.24	94	38	77	83
revB	CACTTATCTGGGACGATCTGCGG	8475–8497	0.32	95	63	66	87
revC	CCACCGCTTGAGAGACTTACTCT	8540–8518	0.25	94	63	37	64

^a^ Double strand break is introduced 3 nt upstream from the underlined SpCas9 PAM sequence. ^b^ Conservation among 97 HIV-1 isolates of six subtypes (A, B, C, D, 01AE, and 02AG) procured from the 2014 sequence compendium (www.hiv.lanl.gov). ^c^ Predicted outcome scores range from 0 to 100, based on algorithms from references described in the text. In silico analysis was done using the CRISPOR tool (crispor.tefor.net). gRNA, guide RNA; PAM, protospacer adjacent motif; MMEJ, microhomology-mediated end-joining.

## Supplementary Materials

The following are available online at https://www.mdpi.com/1999-4915/12/11/1223/s1, Figure S1: GFP-expression measurement as a preliminary assay to determine transduction titer of lentivirus; Figure S2: Cellular growth kinetics of gRNA-Cas9-tranduced MT-4 T cells, compared to mock-infected and Cas9-only-transduced cells; Figure S3: Sequencing analysis of mutations accumulated inside HIV-1 proviral DNA after HIV-1 infection, Cas9 cleavage and NHEJ repair; Figure S4: Colony PCR screening of constructed plasmids. Figure S5: Titer of gRNA-containing lentiviral vectors, produced with co-transfection of gRNA-containing lentiCRISPRv2 with packaging plasmid psPAX2 and VSVG-pseudotyped envelope pHIT/G in Lenti-X 293T cells using FuGENE HD transfection reagent; Figure S6: Kinetics of cell density for selected CRISPR-transduced cells after infection with HIV_NL4-3_ in titers equivalent to 2 ng and 20 ng of p24, as counted using trypan blue exclusion.
