# Supplementary material for: Multiplexed tat-Targeting CRISPR-Cas9 Protects T Cells from Acute HIV-1 Infection with Inhibition of Viral Escape
Source: Viruses. 2020 Oct 28;12(11):1223. doi: 10.3390/v12111223 (PMC7693572; doi:10.3390/v12111223)

viruses

Multiplexed *tat*-targeting CRISPR-Cas9 protects T cell from acute HIV-1 infection with inhibition of viral escape

Supplementary Information

Youdiil Ophinni [1],* Sayaka Miki [2], Yoshitake Hayashi [1] and Masanori Kameoka [2]

[1] Division of Molecular Medicine and Medical Genetics, Kobe University Graduate School of Medicine, 650-0017 Kobe, Japan; icmrt1@gmail.com (Y.H.)
[2] Department of International Health, Kobe University Graduate School of Health Sciences, Hyogo 654-0142, Japan; mkameoka@port.kobe-u.ac.jp (M.K.); cvtokitou@gmail.com (S.M.)
*Correspondence: youdiil_ophinni@yahoo.com (Y.O.)

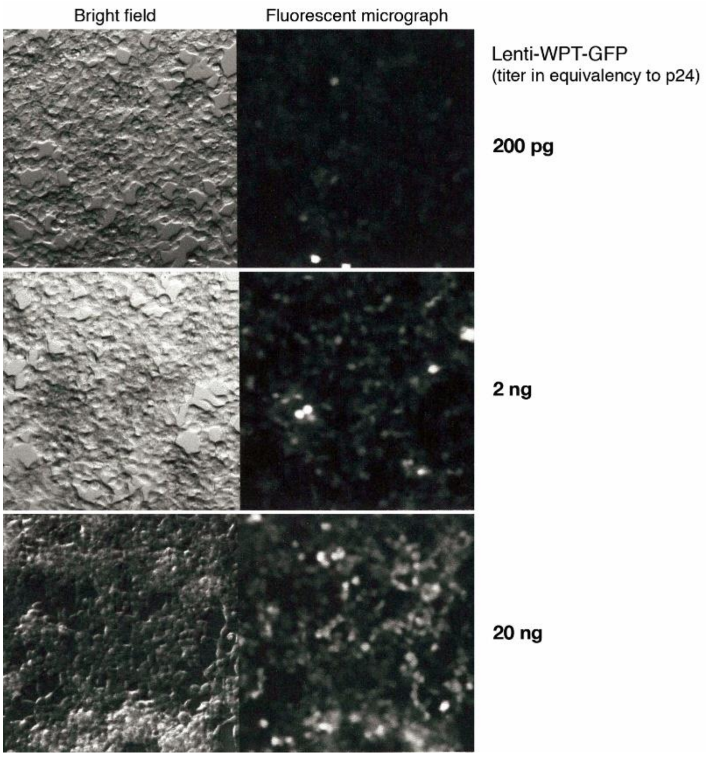

Figure S1. GFP-expression measurement as a preliminary assay to determine transduction titer of lentivirus. GFP-expressing pseudovirus was constructed via co-transfection of pWPT-GFP (Addgene #12255), psPAX2 (Addgene #12260) and VSV-G expressing plasmid pHIT/G. Lentiviral p24 titer was measured using p24 ELISA, p24-titrated viruses were transduced into 293T cells, and GFP expression was visualized 5 days after transduction using fluorescent microscope. Fluorescent micrograph of cells after transduced with 200 pg, 2 ng and 20 ng of lentiviruses are shown. Based on GFP expression, we determined viral titer equivalent to 20 ng of p24 as MOI of 1.

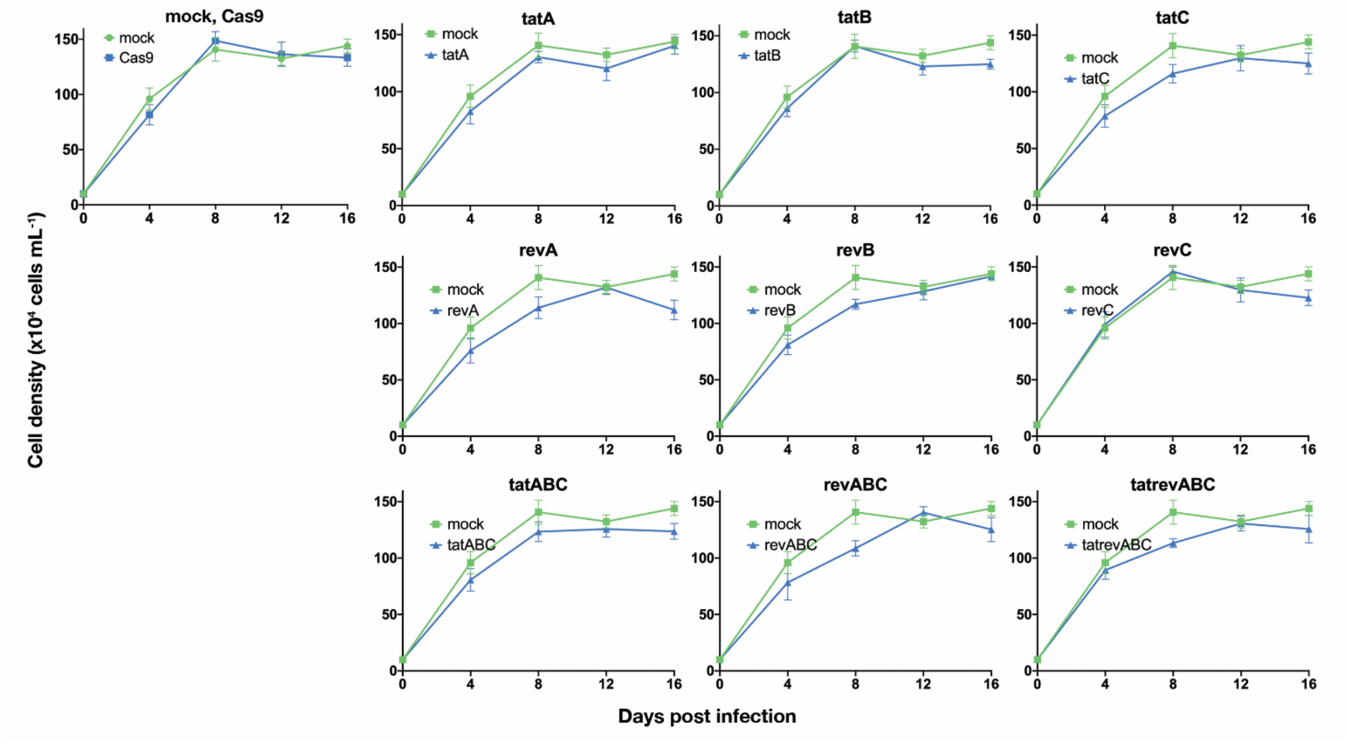

Figure S2. Cellular growth kinetics of Cas9-gRNA-tranduced MT4 T cells, compared to mock-infected and Cas9-only-transduced cells. Cells were seeded at the same density (5x10^4 cells mL^{-1}) in 24-well and cultured in 1 mL of RPMI 1640. Viable cells were counted with trypan blue exclusion. Average values (±SE) of three replicates are shown.

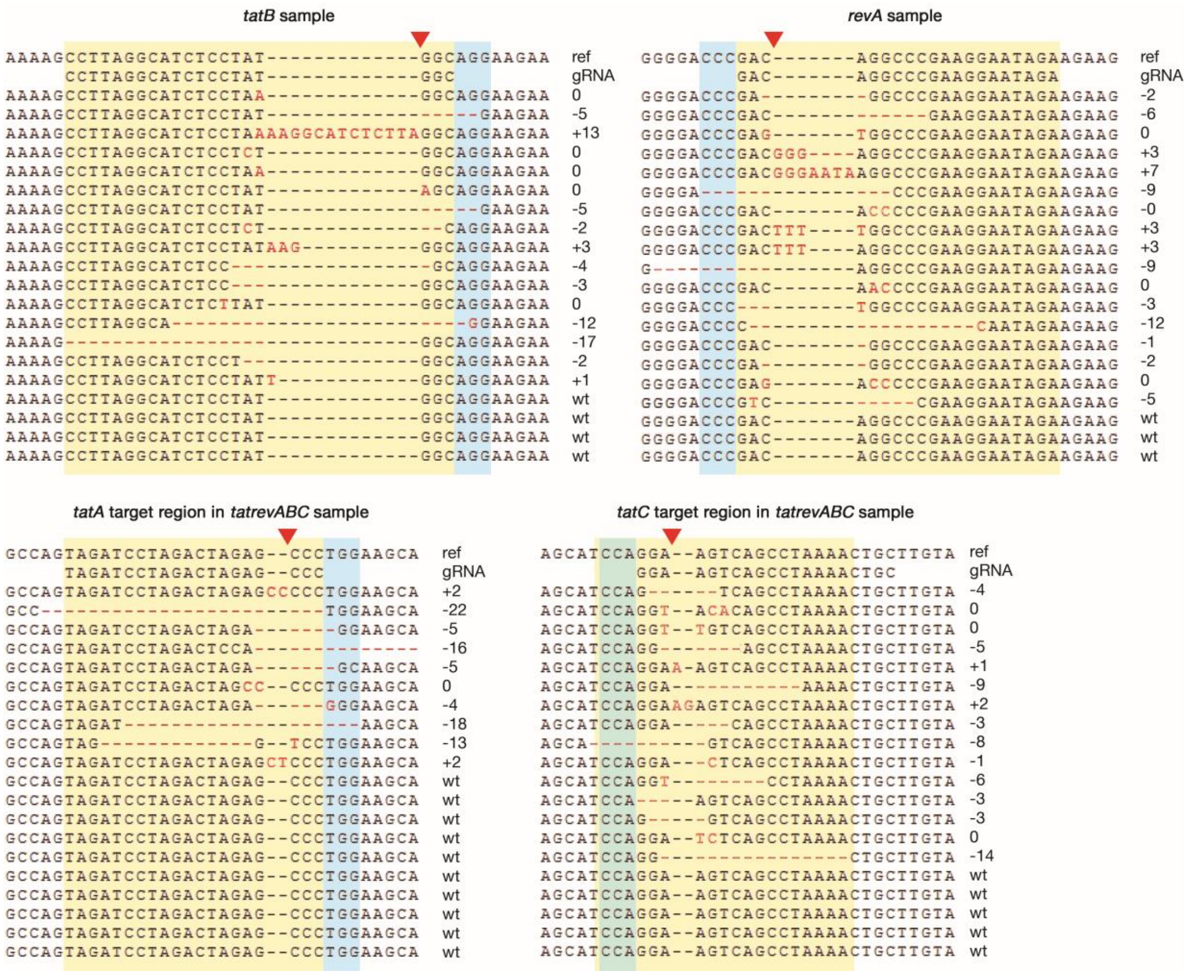

Figure S3. Sequencing analysis of mutations accumulated inside HIV-1 proviral DNA after HIV-1 infection, Cas9 cleavage and NHEJ repair. Cellular DNA was extracted from T cells transduced with *tatB*, *revA*, and *tatrevABC*, 16 days after infection with replicative HIV$_{NL4-3}$. Target gRNA regions were amplified for respective samples, with *tatA* and *tatC* regions amplified from *tatrevABC*-transduced cells. Amplicons were cloned using TA cloning vector to produce 20 samples for each gRNA target, Sanger sequenced and aligned with reference (topmost). Summary of results were shown in Figure 3. Target gRNA and PAM sequence were shown in yellow and blue box, respectively. Mutations indicated in red and Cas9-cutting site noted under the red triangles. Numbers to the right of sequences signify changes in sequence length after mutation; wt indicated wild-type, non-mutated sequences.

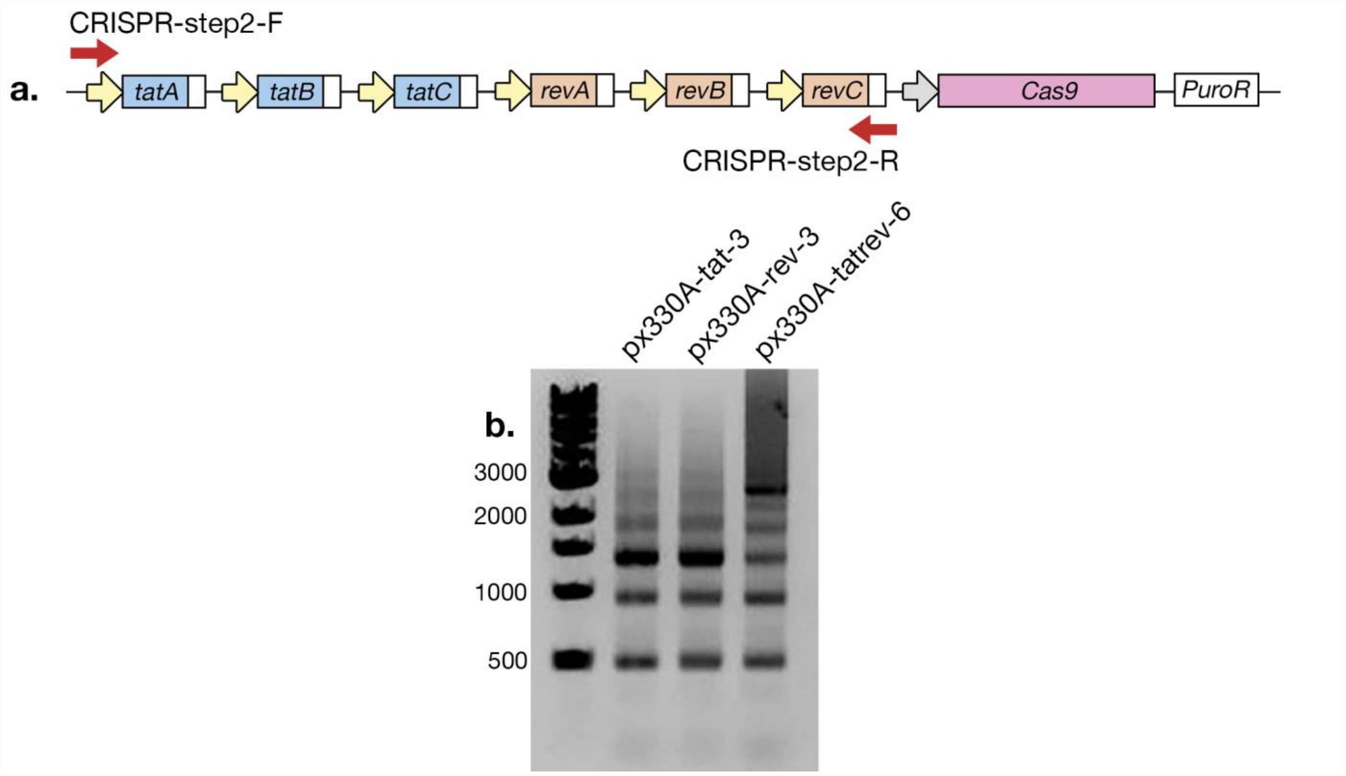

Figure S4. Colony PCR screening of constructed plasmids. (**a**) Schematic for the PCR amplification, shown for px330A-tatrev-6 as example. Amplicon stretched from immediate upstream of the first gRNA to immediate downstream of the last gRNA; amplification was done using primers as indicated. (**b**) Gel electrophoresis of amplicons. Number of bands indicate the number of gRNAs contained in the assembled clones.

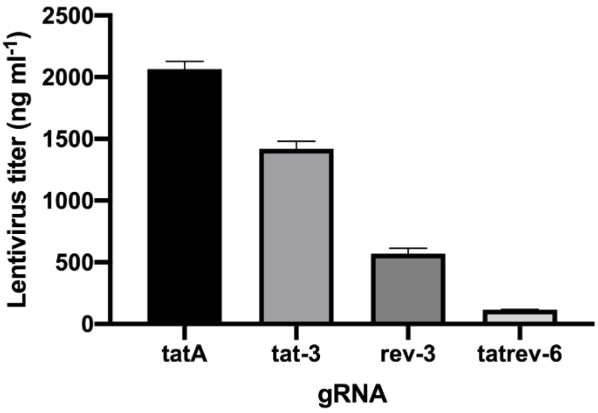

Figure S5. Titer of gRNA-containing lentiviral vectors, produced with co-transfection of gRNA-containing lentiCRISPRv2 with packaging plasmid psPAX2 and VSVG-pseudotyped envelope pHIT/G in Lenti-X 293T cells using FuGENE HD transfection reagent. Lentiviruses were harvested three days after transfection. Titers of the all-in-one multiplexed gRNA vectors are shown, with that of single gRNA *tatA* shown as comparison. Average values (±SE) of three replicates are shown.

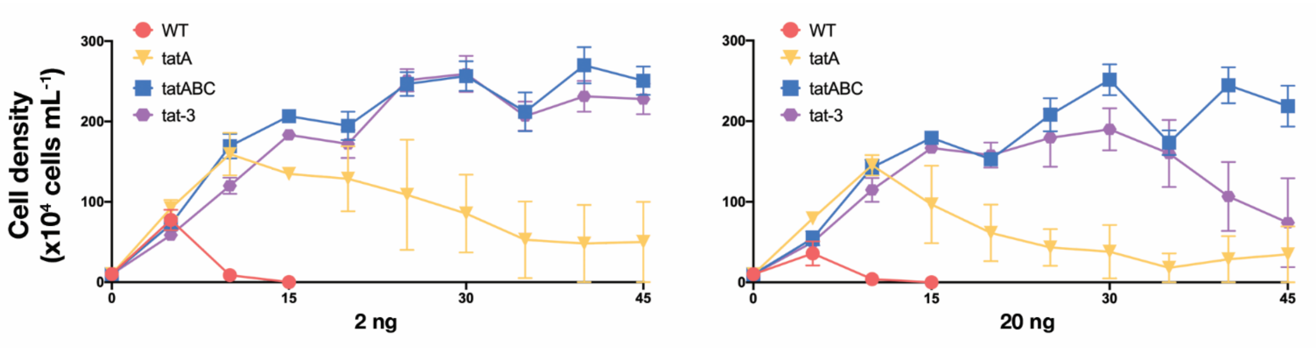

Figure S6. Kinetics of cell density for selected CRISPR-transduced cells after infection with HIV_{NL4-3} in titers equivalent to 2 ng (left) and 20 ng (right) of p24, as counted using trypan blue exclusion. All WT cells died from 15 days onwards. Half of cell culture were passaged every 15 days. Assays were performed three times with average values (±SE) as shown.

Uncropped blot and gel images

Original Western Blot images of Figure 1b

Original uncropped Western blot images of Figure 1b. Figures were not doctored or joined together from different photographs. Blotted PVDF membranes were incubated with anti-Cas9 (top) and anti-beta-actin (bottom) as loading control.

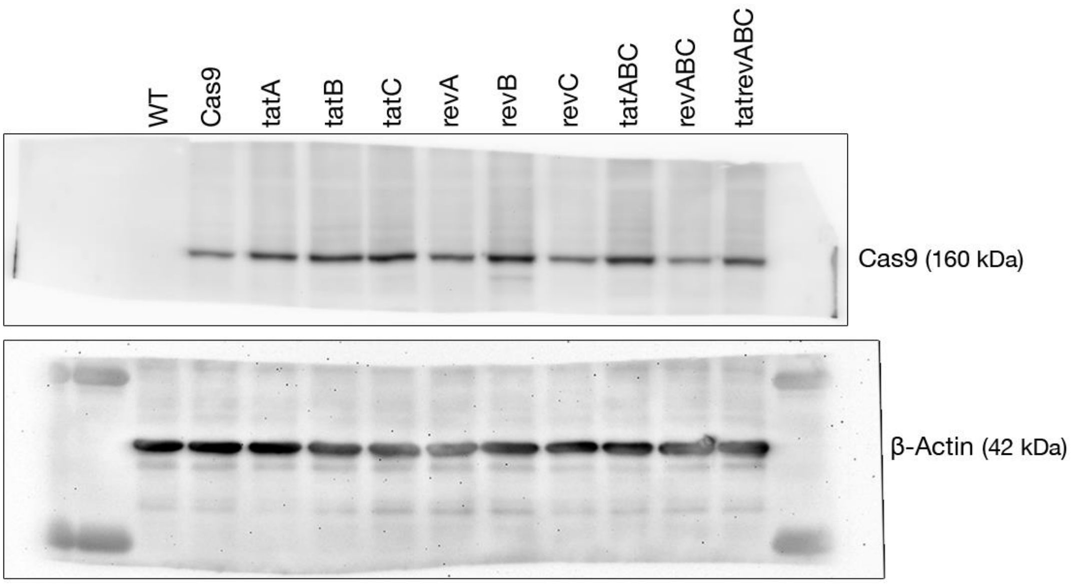

Original electrophoresis gel image of Figure 2b

Original uncropped gel images of Figure 2b. Gel image were not doctored or joined together from different photographs.

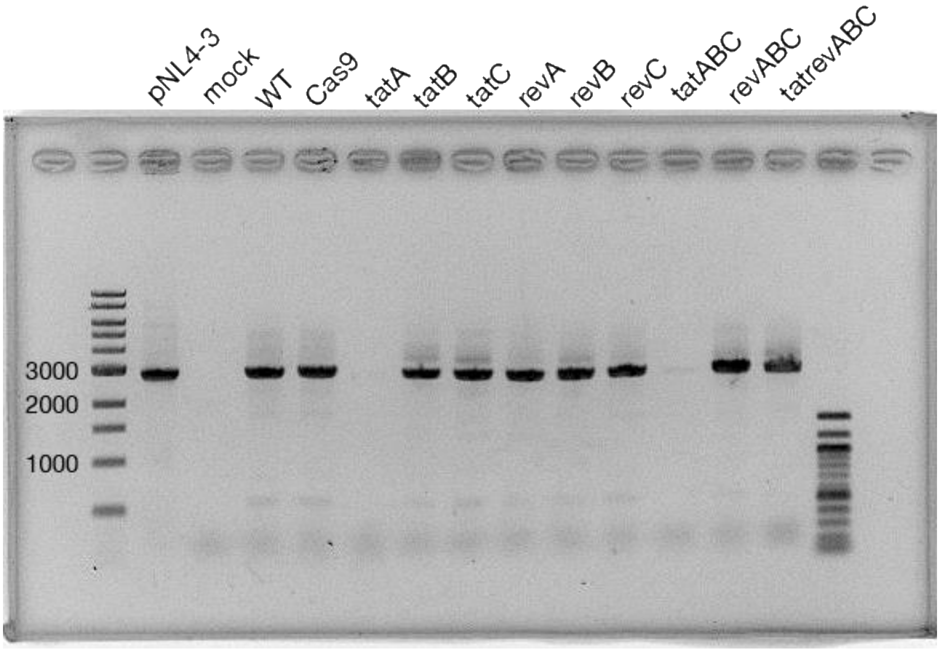

Original Western Blot images of Figure 5b

Original uncropped Western blot images of Figure 5b. Membrane images were not doctored or joined together from different photographs. Blotted PVDF membranes were incubated with anti-FLAG (top) and anti-beta-actin (bottom).

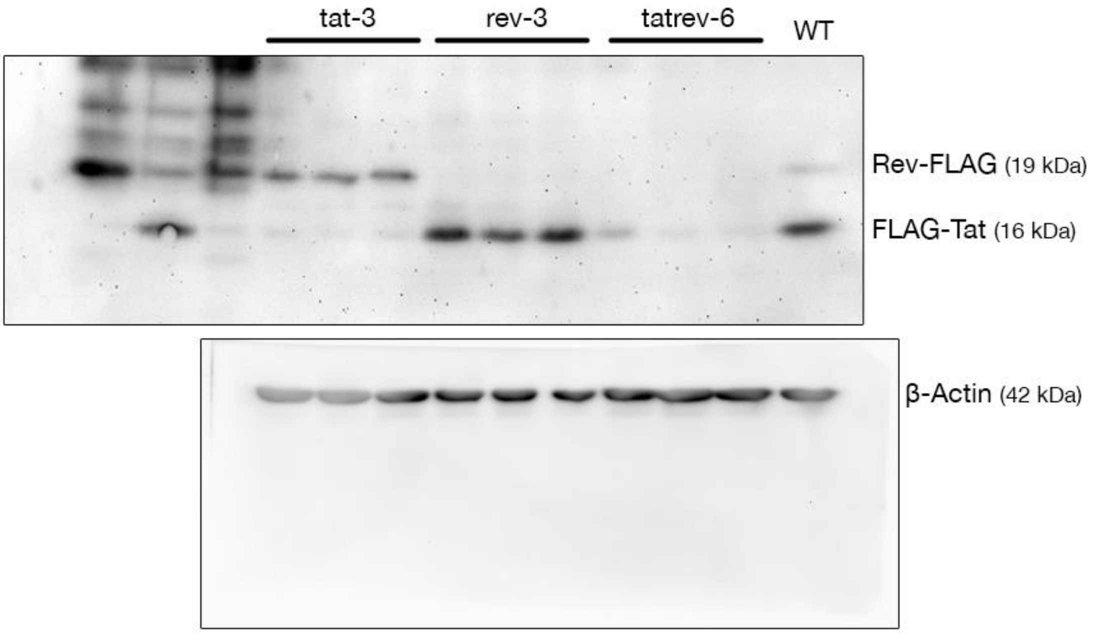

Original electrophoresis gel image of Figure S4

Original uncropped gel images of Figure S4. Figures were not doctored or joined together from different images.

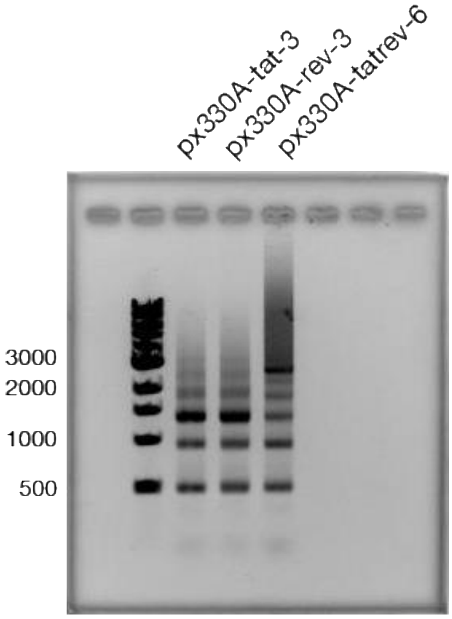

Supplement: Supplementary file 1 [file viruses-12-01223-s001.pdf]
